# Effects of early-life environmental stress on risk-taking tendency of adolescents in rural areas of southwestern China

**DOI:** 10.3389/fpsyt.2024.1520790

**Published:** 2024-12-12

**Authors:** Jing Wu, Qiaobing Wu

**Affiliations:** ^1^ Department of Early Childhood Education, School of Vocational and Technical Education, Shenzhen Polytechnic University, Shenzhen, Guangdong, China; ^2^ Department of Applied Social Sciences, The Hong Kong Polytechnic University, Hong Kong, Hong Kong SAR, China

**Keywords:** risk-taking, life history theory, early-life stress, left-behind, adolescents

## Abstract

**Introduction:**

Adolescence is a critical developmental phase characterized by increased risk-taking behaviors, which are not inherently maladaptive. According to life history theory, individuals raised in harsh and unpredictable environments are more likely to adopt faster life history strategies, favoring immediate rewards over long-term benefits. Yet, limited empirical research explore the psychological mechanism about how early-life environmental stresses influence adolescents’ risk-taking. In rural China, left-behind children face economic and social vulnerabilities due to parental migration to urban areas for employment. This study’s first goal was to identify the specific elements of early-life environmental stresses that impact adolescents’ risk-taking tendencies from a developmental evolutionary perspective. The second goal was to construct and test a synthesized model of how objective and subjective environmental stresses influence adolescents’ risk-taking.

**Methods:**

A total of 610 middle school students in rural China completed questionnaires assessing early-life environmental stresses and risk-taking tendencies. The sample included 318 left-behind adolescents, 120 single-left-behind adolescents with one parent, and 138 non-left-behind adolescents. Structural equation modeling tested the hypothesized model, examining direct and indirect effects of environmental stresses on risk-taking.

**Results:**

Objective early-life environmental stresses, such as low socioeconomic status (SES), high mortality cues, and high mobility cues, predicted faster life history strategies, marked by shorter future orientation and increased risk-taking tendencies. Subjective perceptions of environmental unpredictability and parental warmth mediated the influence of SES on risk-taking. Biological sensitivity moderated mortality cues’ influence on perceived parental warmth. Sense of control failed to mediate the relationship between early-life stresses and risk-taking. Left-behind adolescents experienced more mobility and mortality cues, perceived greater unpredictability, and reported less parental warmth than their peers. Despite no significant difference in overall risk-taking, left-behind adolescents exhibited higher health/safety risk-taking tendencies.

**Discussion:**

This study provides a comprehensive model linking early-life environmental stresses to adolescents’ risk-taking, integrating objective and subjective measures of stress. The findings offer insights into mechanisms driving risk-taking tendencies. Also, it have significant implications for developing interventions aimed at mitigating the adverse effects of early-life stress on adolescent development, particularly for left-behind children in rural China.

## 1 Introduction

Adolescence, especially middle adolescence that spans the age of 13 to 17, is widely recognized as a developmental period marked by the emergence and escalation of risk-taking behaviors, such as delinquency, drug use, crime, and unprotected sex activities (e.g., 1, 2). Considering the significant challenges and potential dangers associated with such behaviors, the prevailing developmental psychopathology framework regards them as maladaptive and disturbed developmental outcomes arising from stressful childhood life experiences together with personal or biological vulnerabilities (3, 4). But indeed, many life decisions involve a balance between anticipated rewards against potential losses. Risk-taking behaviors are often linked to potentially desirable outcomes, such as short-term pleasure, immediate gratification, and the potential for substantial gains, despite inherent risks (5). From an evolutionary standpoint, risky behaviors are thought to be conditionally adaptive when anticipated benefits outweigh the potential costs (6, 7).

According to life history theory (8, 9), individuals respond to the environmental contexts by developing distinct phenotypes tailored to those environments. Life history strategies are believed to exist along with a slow-to-fast continuum (8). Individuals following a “slow life history strategy” tend to prioritize long-term planning and future rewards, exhibiting characteristics such as delayed reproduction, later physical maturation, commitment to long-term relationships, and low levels of risk-taking (10). In stark contrast, those adopting a “fast LH strategy” focus intensely on immediate gratifications rather than future gains. They are more preoccupied with mating opportunities and related activities, demonstrating early physical maturation, high mating effort, short-term mating strategies, and increased risk-taking behaviors.

Childhood environments significantly influence whether an individual adopts a fast or slow life history strategy, with fast strategies often emerging in stressful early environments (11, 12). Ellis et al. Emphasized two key environmental parameters—harshness and unpredictability—that uniquely influence life history strategies (13). “Harshness” refers to environmental conditions that increase mortality and morbidity, such as resource scarcity, pathogen prevalence, extreme climates, and predator threats. “Unpredictability”, on the other hand, describes the variability and randomness in the environment that challenge the consistent application of adaptive strategies. Both compel organisms to adopt faster life history strategies, promoting early maturation and reproduction to maximize survival in adverse conditions (14). A longitude research conducted by Gardner and Steinberg (15) demonstrated that adolescents experiencing higher familial and ecological stress in the 6th grade experienced earlier sexual debut and greater sexual risk-taking by the 12th grade. Prior studies have predominantly examined the impact of harshness and unpredictability on life history strategies collectively (14–17), with scant attention to their distinct impacts. Martínez et al. found that people who experienced more unpredictability in their childhood tended to set goals on relatively shorter time horizons (18). Brumbach et al. (19) found that greater unpredictability (indicated by self-reported exposure to violence from conspecifics) and harshness (measured by frequent changes or ongoing inconsistency in several dimensions of childhood environments) during adolescence independently predicted the adoption of faster life history strategies. Belsky (20) observed that unpredictability alone, rather than harshness, predicted a higher number of sexual partners by age 15, although this was assessed by examining a single life history outcome. His work revealed that unpredictable environments during early childhood correlate with more sexual partners by age 15, mediated by maternal depressive symptoms and sensitivity. However, existing measures of harshness and unpredictability are limited. For instance, childhood SES is often used to denote environmental harshness, yet it lacks comprehensiveness regarding morbidity-mortality levels (13). Therefore, one objective of this study was to delineate how specific aspects of environmental harshness and unpredictability influence the adoption of slower versus faster life strategies.

Based on the report titled “What the 2020 Census Can Tell Us About Children in China: Facts and Figures” published by the National Bureau of Statistics, the number of school-age left-behind children and adolescents in China was approximately 66.93 million as of 2020 (21). The report highlighted an imbalanced geographic distribution, with the majority residing primarily in the Midwest and Southwest regions of China. Numerous studies have compared the emotional and behavioral problems between left-behind children and non-left-behind children (22–24). Sun et al. found that left-behind children generally report lower life satisfaction, diminished self-esteem, and increased levels of depression compared with non-left-behind children (24). Factors alleviating these issues include the presence of one parent, more frequent parental contact, and a shorter duration since parental migration. In this study, rural adolescents cared for by a single parent, grandparents, or other relatives, due to parental migration for work, are termed left-behind adolescents. Parental absence contributes to uncertain feeling about the external world. By splitting the elements of harshness and unpredictability, this study examined their distinct influences on risk-taking tendencies, focusing on left-behind versus non-left-behind groups.

Developmentally oriented evolutionary psychologists emphasize the interactions among gene, environment, and development (25, 26), highlighting the necessity of considering both objective conditions and subjective perceptions. Evidence suggests that adverse rearing environments particularly impact children considered temperamentally or genetically “vulnerable,” characterized by heightened sensory-processing sensitivity to both adverse and supportive contexts, as described by Aron and Aron (27). The differential susceptibility hypothesis (28, 29) proposes that context-sensitive individuals undergo significant developmental changes based on environmental influences. Given that the concepts of objective and subjective environmental stresses are distinguished, this study considered biological sensitivity a moderating factor between environmental stresses and subjective perceptions. Highly sensitive individuals, viewed as more “vulnerable” to negative events, are expected to perceive greater unpredictability in high-stress contexts than their less sensitive counterparts.

Differences in childhood environments shape how individuals perceive threats as either extrinsic or intrinsic. Mittal and Griskevicius (30) demonstrated that feelings of uncertainty alter people’s sense of control over their environment, with those from disadvantaged backgrounds exhibiting reduced sense of control. Correlational studies further suggest a direct link between SES and sense of control, with lower SES associated with a decreased sense of control (31, 32). This study hypothesized that exposure to environmental stresses, including high unpredictability and low SES, leads individuals from economically disadvantaged childhoods to experience a diminished sense of control compared to their wealthier peers.

**Figure 1:**
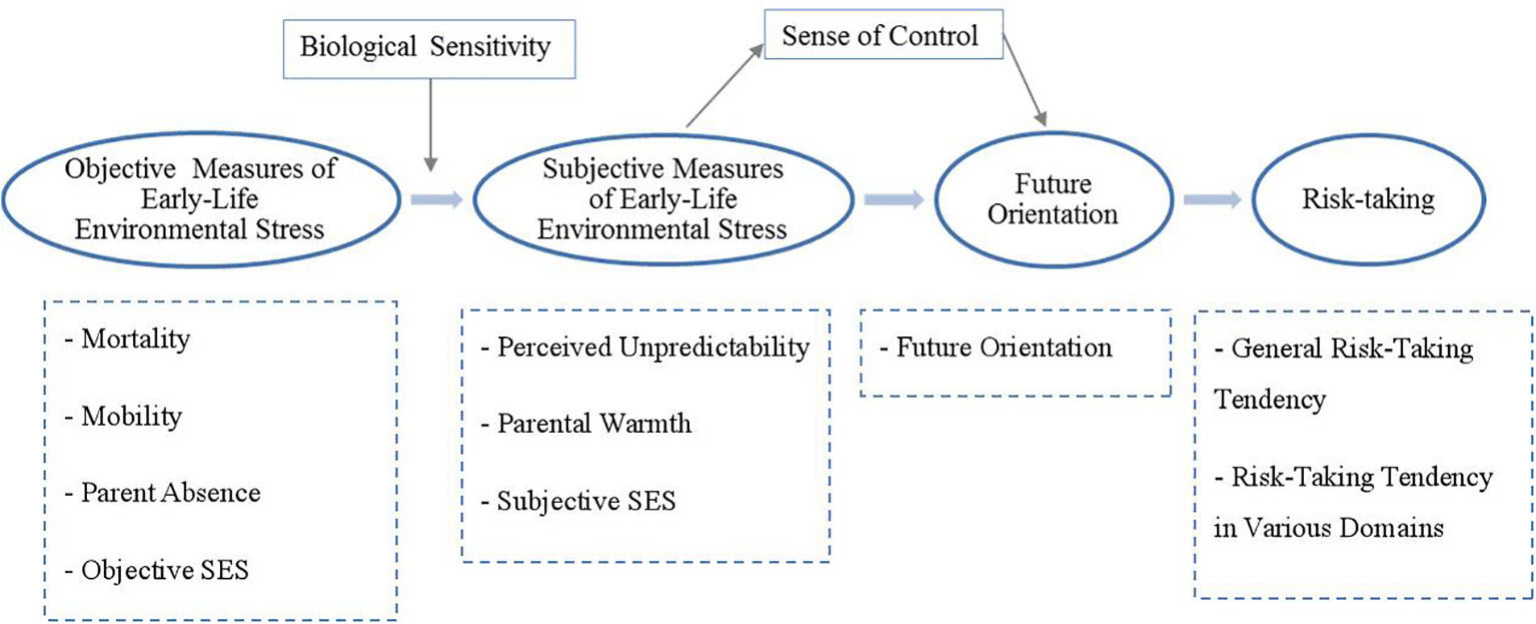
Research hypothesis model.

Building on the preceding theoretical framework and existing literature, the main purpose of this study was to assess how the measured early childhood environment stresses are associated with risk-taking perception and behaviors. Besides, some intermediate variables were also considered when examining the mechanisms, including biological sensitivity to the environment, subjectively perceived unpredictability, perceived parental warmth, sense of control, and future orientation. Lastly, the affecting model comparing left-behind and non-left-behind groups was also performed. The research hypothesis model, as illustrated in **Figure 1**, outlines how early-life environmental stress and individual characteristics influence adolescents’ risk-taking.

## 2 Materials and methods

### 2.1 Participants

A rural county in Chongqing Municipality, known for its substantial population of rural left-behind children, was selected to conduct the survey. A total of 716 junior and senior secondary school students aged between 10-19 years was recruited to complete the questionnaire. Students completed the questionnaires anonymously during class. They were informed of their rights to ask questions and withdraw from the study at any time. Of these participants, 610 provided valid responses, resulting in an effective response rate of 85.19%. **Table 1** outlines the distribution of participants: 347 (56.88%) were male and 263 (43.11%) were female. Regarding their left-behind status, 141 (23.11%) students were non-left-behinds, 121 (19.83%) were single-left-behinds living with one parent, and 316 (51.80%) were left-behinds with neither parent around. An additional 34 students were classified as “others”— due to parental absence for reasons such as such as death, divorce, illness, incarceration, or business trips, rather than migrant employment.

**Table 1:** Distribution of participants.

Grade	Gender	Left-behinds	Single-Left-behinds	Non-left-behinds	Others	Total
7	Male	47 (7.7%)	17 (2.8%)	22 (3.6%)	2 (0.3%)	88 (14.4%)
	Female	27 (4.4%)	12 (2.0%)	12 (2.0%)	1 (0.2%)	52 (8.5%)
8	Male	40 (6.6%)	15 (2.5%)	24 (3.9%)	6 (1.0%)	85 (13.9%)
	Female	30 (4.9%)	8 (1.3%)	16 (2.6%)	4 (0.7%)	58 (9.5%)
10	Male	43 (7.1%)	21 (3.4%)	17 (2.8%)	4 (0.7%)	85 (13.9%)
	Female	49 (8.0%)	21 (3.4%)	12 (2.0%)	6 (1.0%)	88 (14.4%)
11	Male	51 (8.4%)	14 (2.3%)	21 (3.4%)	3 (0.5%)	89 (14.6%)
	Female	31 (5.1%)	12 (2.0%)	14 (2.3%)	8 (1.3%)	65 (10.7%)
Total	Male	181 (29.7%)	67 (11.0%)	84 (13.8%)	15 (2.5%)	347 (56.9%)
	Female	137 (22.5%)	53 (8.7%)	54 (8.9%)	19 (3.1%)	263 (43.1%)
	Total	318 (52.1%)	120 (19.7%)	138 (22.6%)	34 (5.6%)	610 (100.0%)

The study involving humans was approved by Survey and Behavioral Research Ethics of The Chinese University of Hong Kong. The study was conducted in accordance with the local legislation and institutional requirements. The participants provided their written informed consent to participate in this study.

### 2.2 Measures

All measures in this study were administered in simplified Chinese. Measures assessing mortality cues, mobility cues, and parent absence were self-developed by researchers, while other measures were translated and modified as necessary.

#### 2.2.1 Mortality cues

Mortality cues were defined as personal experiences related to death or traumatic events (33). Participants reported specific childhood occurrences including the death of cohabiting family members, serious injury or illness, and relevant disasters or accidents. A response indicating “never” was scored as 0, while any experience of the events was scored as 1; a parent’s death received a score of 2. The total score for mortality cues was calculated by summing the scores across all reported events.

#### 2.2.2 Mobility cues

Mobility cues represented objective environmental unpredictability in this study. Three specific indicators were included: household moves, parental divorce, and school transfers. A response of “never” indicating no occurrence of these events, was scored as 0, while any experience of these events was scored as 1. The total score for mobility cues was the sum of the three indicators.

#### 2.2.3 Socioeconomic status

Due to many adolescent participants being unaware of their parents’ income, a subjective measure of SES was used (34). Participants rated their agreement with four statements: “My family usually had enough money for things when I was growing up”, “I grew up in a relatively wealthy neighborhood”, “My parents were often anxious about not having enough money for daily life”, and “I felt relatively wealthy compared to other kids in my school”, using a 6-point Likert scale. The Cronbach’s alpha for this scale was. 926.

#### 2.2.4 Parent absence

A self-designed parent-absence timetable was provided for participants to complete. They documented each extended absence (over three months) with details including “who left (father/mother/both parents)”, “reason for leaving”, “my age at that time”, “duration of absence”, “whom I lived with during the absence”, and “how often my parent(s) contacted me (times per week)”. Individuals who reported parental absence due to “work far from hometown” were categorized as “left-behind”, while others were categorized as “non-left-behind”. Participants selected their household status from eight options, ranging from “non-absence, both parents present” to various forms of absence, including living with relatives or alone. This was analyzed to differentiate between “non-left-behinds”, “single-parent-left-behinds”, and “left-behinds” with parents’ absence.

#### 2.2.5 Perceived unpredictability

Subjective environmental unpredictability was assessed using subscales from the Family Unpredictability Scale (R-FUS) (35), which were revised for this study from a child’s perspective. Adolescents reported their daily life experiences across 15 items from three dimensions: meals (e.g., “The dining location varies occasionally for me”), illness (e.g., “I recover very quickly every time I get ill or hurt”), and financial situations (e.g., “I borrow money from my classmates from time to time”). Responses were recorded on a 6-point scale, with 1 indicating “not at all matches me” and 6 indicating “very true of me”. The Cronbach’s alpha for this scale was. 718.

#### 2.2.6 Parental warmth

Parental warmth was measured using a revised scale based on the parental support subscale of the Children’s Report on Parent Behavior Inventory (CRPBI) (36, 37) and the discipline subscale of the R-FUS (35). This combined a 10-item scale featuring five items focused on support (e.g., “My parents have enough time to care for me every day”) and five items focusing on discipline (e.g., “How my parents will act in a specific discipline situation depends on their moods”). The Cronbach’s alpha for this scale was. 847.

#### 2.2.7 Future orientation

Future orientation was assessed using a 15-item self-report scale (38) designed to gauge adolescents’ outlook on the future. Participants evaluated their agreement with statements about themselves across three dimensions: time perspective (e.g., “I always think about what tomorrow will bring”), planning ahead (e.g., “I find that breaking big projects down into small steps isn’t really necessary”), and anticipation of consequences (e.g., “I believe it’s beneficial to think through potential outcomes before making a decision”). Participants rated each statement on a 6-point scale, ranging from 1 (“totally not true for me”) to 6 (“totally true for me”). Responses for negatively worded items were reverse scored and averaged, with higher scores indicating a stronger future orientation. The internal consistency for this scale was moderate (alpha = .787).

#### 2.2.8 Biological sensitivity

Biological Sensitivity was measured using the Highly Sensitive Person Scale (27), a 17-item questionnaire that has demonstrates strong reliability as well as convergent and discriminant validity. Example items include, “Are you made uncomfortable by loud noises?” and “Are you particularly sensitive to the effects of caffeine?” The sensitivity score was treated as a continuous variable, with an internal consistency of acceptable reliability (alpha = .826).

#### 2.2.9 Sense of control

The sense of control was assessed using an established four-item measure from Rodin (39). Participants indicated their level of agreement with statements such as: (a) I can achieve anything I set my mind to; (b) my future mostly depends on my own actions; (c) when I truly want to accomplish something, I usually find a way to succeed; and (d) whether I achieve my goals is largely within my control. Responses for each item were provided on a 6-point scale (1 = strongly disagree, 6 = strongly agree). Responses were given on a 6-point scale (1 = strongly disagree, 6 = strongly agree) and aggregated across the four items. The internal consistency of this measure was moderate (alpha = .765).

#### 2.2.10 Risk taking

The Domain-Specific Risk-Taking (DOSPERT) scale developed by Blais and Weber (40) was utilized to assess risk-taking behaviors across five domains: financial, health/safety, ethical, recreational, and social interaction. The Chinese version of DOSPERT was validated in previous studies by Hu and Xie (41), which confirmed the preservation of similar domains within the Chinese context. In the current study, confirmatory factor analysis (CFA) showed acceptable data-model fit (*df* = 270, *χ^2^* = 1332.82; *CFI* = .851; *TLI*= .835; *RMSEA* = .080; *SRMR* = .061). The internal consistency for this scale was also acceptable (alpha = .873).

### 2.3 Data analysis

All statistical analyses were carried out using SPSS 20.0 and Mplus 7.0.

The analysis was organized into two main parts: the first part comprised descriptive analyses, which included summary statistics and examinations of the relationships between risk-taking behaviors and their proposed antecedents, as well as comparisons of the variables among three groups: left-behinds, single-parent left-behinds, and non-left-behinds.

The second part employed structural equation modeling (SEM) to examined the proposed mechanisms by which these antecedents, mediators and moderators influence adolescents’ risk-taking tendencies. Preliminary analyses were performed to evaluate correlations among demographic variables, predictors, and outcome variables.

## 3 Results

### 3.1 Descriptive statistics

**Table 2** presents the means, standard deviations, and correlations of the variables for the entire sample. The data indicate that most associations between the antecedents and adolescents’ risk-taking tendencies were statistically significant, with correlation coefficients ranging from *r* = .003 to -.420. Overall risk-taking tendencies were positively associated with age, mortality cues, perceived unpredictability, and biological sensitivity. Conversely, risk-taking was negatively correlated with subjective socioeconomic status (SES), parental warmth, and future orientation.

**Table 2:** Means, standard deviations, and correlations of the variables.

		M (SD)	1	2	3	4	5	6	7	8	9	10
**1**	Age	15.28 (1.73)										
**2**	Left-behind type	1.69 (.83)	-.100 ^*^									
**3**	Mobility	1.47 (.74)	.198 ^**^	-.132^**^								
**4**	Mortality	.89 (.70)	.154^**^	-.149^**^	.111^**^							
**5**	Subjective SES	4.41 (.97)	-.218^**^	.150^**^	-.042	-.071						
**6**	Perceived unpredictability	2.72 (.79)	.065	-.092 ^*^	.036	.078	-.410^**^					
**7**	Parental warmth	3.97 (.87)	-.122^**^	.150^**^	-.119^**^	-.055	.013	-.416^**^				
**8**	Biological sensibility	2.20 (.71)	.048	-.008	.048	.035	-.344^**^	.437^**^	.131^**^			
**9**	Sense of control	3.62 (1.16)	.182^**^	.008	.016	-.025	-.242^**^	.316^**^	.153^**^	.311^**^		
**10**	Future orientation	3.74 (.85)	-.094 ^*^	.021	-.028	.053	-.075	-.324^**^	.340^**^	.135^**^	.251^**^	
**11**	Risk taking	2.20 (.71)	.216^**^	.017	.003	.099 ^*^	-.420^**^	.516^**^	-.193^**^	.351^**^	.342^**^	-.108^**^

*N* = 610; ^*^ *p* <.05; ^**^ *p* <.01; left-behind type: 1= left-behinds with neither parent around, 2 = left-behinds with either parent, 3 = non-left-behinds.

The variable representing left-behind type (with 1 for left-behinds with neither parent present, 2 for single-left-behinds, and 3 for non-left-behinds) showed a negative relationship with mobility cues, mortality cues, and perceived unpredictability while positively correlating with subjective SES and parental warmth. Mobility cues were negatively related to parental warmth. Furthermore, subjective SES was positively associated with a sense of control and negatively associated with perceived unpredictability, biological sensitivity, and general risk-taking.

One-way ANOVAs were conducted to compare the characteristics of adolescents across different left-behind types, excluding participants with special left-behind circumstances from this analysis. As demonstrated in **Table 3**, adolescents in the left-behind, single-left-behind, and non-left-behind groups exhibited significant differences in mobility cues (*F*(2, 573) = 5.08, *p*<.01) and mortality cues (*F*(2, 573) = 4.49, *p*<.05). Additionally, they reported different subjective SES levels (*F*(2, 573) = 7.10, *p* <.01), and perceived significant differences in unpredictability (*F*(2, 573) = 2.87, *p* <.05) and parental warmth (*F*(2, 573) = 6.81, *p* <.01).

**Table 3:** Means and standard deviations of the variables among Left-Behinds, Single-Left-Behinds, and Non-Left-Behinds.

	Left-Behinds (*N* = 318) *M (SD)*	Single-Left-Behinds (*N* = 120) *M (SD)*	Non-Left-Behinds (*N* = 138) *M (SD)*	*F*
Mobility	1.49 (.71)	1.14 (.69)	.95 (.74)	5.08^**^
Mortality	1.14 (.95)	1.09 (.92)	.86 (.96)	4.49^*^
Subjective SES	4.32 (.99)	4.40 (.84)	4.68 (.94)	7.10^**^
Perceived unpredictability	2.78 (.69)	2.63 (.67)	2.60 (.71)	2.87^*^
Parental warmth	3.54 (1.16)	3.77 (1.05)	4.11 (.86)	6.81^**^
Biological sensibility	3.40 (.81)	3.41 (.88)	3.39 (.89)	.04
Sense of control	3.61 (1.13)	3.55 (1.08)	3.64 (1.26)	1.00
Future orientation	3.74 (.88)	3.74 (.80)	3.78 (.84)	.17
Risk taking (Total Score)	2.20 (.67)	2.11 (.65)	2.24 (.80)	2.24
Social	2.96 (.94)	2.83 (.89)	3.10 (1.06)	2.44
Ethical	2.00 (.84)	2.04 (.83)	2.07 (.95)	1.23
Health/Safety	2.25 (.84)	2.03 (.83)	2.04 (.93)	2.81^*^
Financial	1.82 (.77)	1.66 (.67)	1.86 (.88)	2.54
Recreational	2.10 (1.00)	1.66 (.67)	1.86 (.88)	.98

^*^ *p* <.05; ^**^ *p* <.01.

The LSD *post hoc* tests revealed that left-behinds reported significantly more mobility cues than non-left-behind peers (contrast estimates = 3.45; *p* <.01). Both left-behinds and single-left-behinds reported experiencing more mortality cues compared to non-left-behinds (contrast estimates = 2.95 and 2.02, respectively; *p* <.01 and *p* <.05). Regarding socioeconomic status, non-left-behinds reported higher subjective SES than single-left-behinds (contrast estimates = 2.53; *p* <.05).

Further results from the LSD *post hoc* tests indicated that left-behinds perceived significantly greater unpredictability than both single-left-behinds and non-left-behinds (contrast estimates = 1.86 and 2.01, respectively; *p* <.05). Additionally, non-left-behinds reported significantly higher parental warmth than single-left-behinds (contrast estimate = 2.28; *p* <.01), and single-left-behinds perceived more parental warmth than left-behind peers (contrast estimate = 1.91; *p* <.05).

While there were no significant differences in total risk-taking scores among the different groups, a notable difference was observed in risk-taking behaviors related to health and safety (*F*(2, 573) = 2.81, *p* <.05). Left-behind adolescents reported taking more risks in the health/safety domain than both single-left-behind and non-left-behind adolescents (contrast estimates = 1.80 and 1.79, respectively; *p* <.05).

### 3.2 The structure equation model

To analyze the relationships among the variables in this study, structural equation modeling (SEM) was employed. The model posited that mobility cues, mortality cues, and subjective SES would predict both future orientation and risk-taking, with perceived unpredictability and parental warmth serving as mediators. Biological sensitivity was omitted from the model because its inclusion of mediating role diminished the overall fit. The fitness indices supported the model, indicating a satisfactory fit to the data (*df* = 7, *χ^2^* = 29.39; *CFI* = .944; *TLI*= .815; *RMSEA* = .076; *SRMR* = .034).

**Figure 2:**
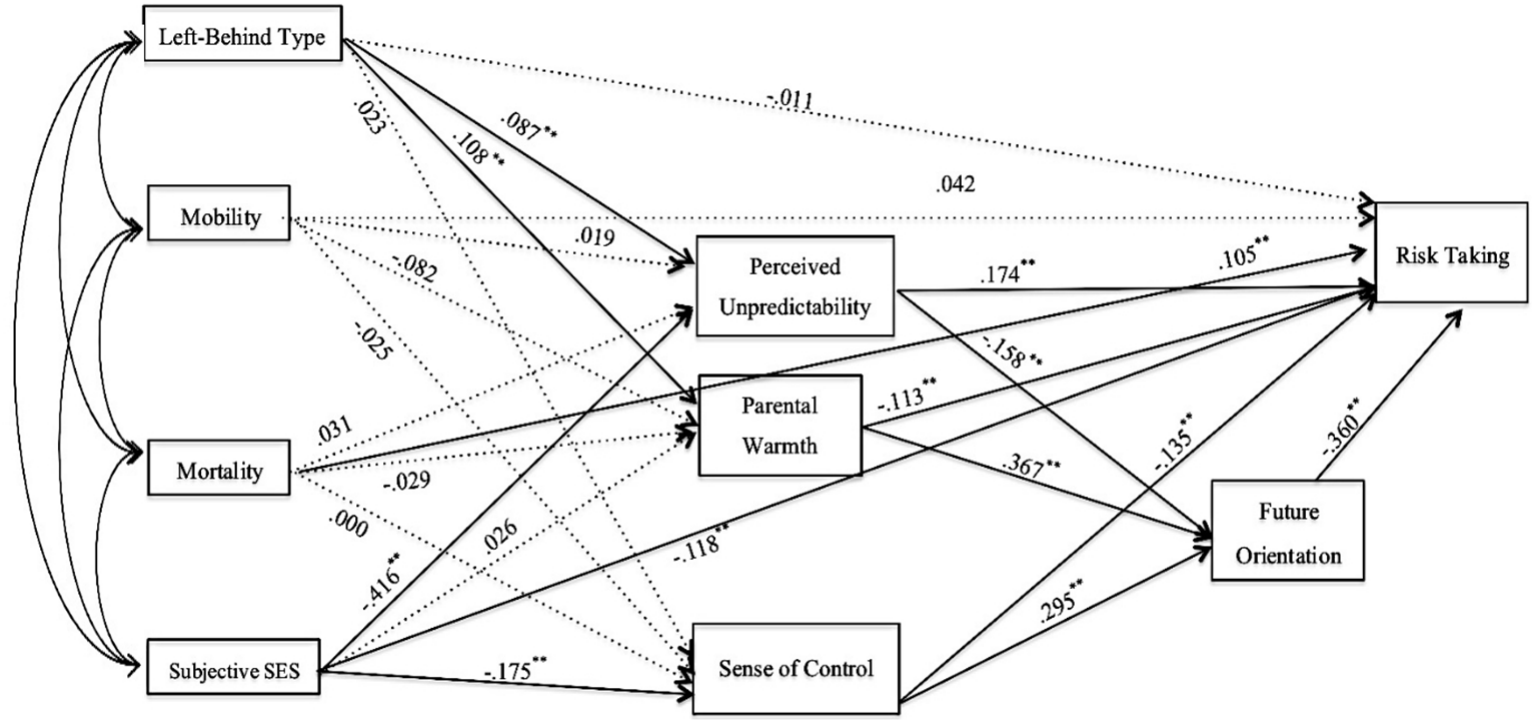
The model of SEM analysis.

As illustrated in **Figure 2**, age, mortality cues, and subjective SES directly influenced risk-taking behaviors. Perceived unpredictability acted as a mediator between subjective SES and both future orientation and risk-taking, while parental warmth mediated the relationship between mobility cues and these two outcomes. Notably, subjective SES was found to negatively predict a sense of control, which in turn positively predicted future orientation and risk-taking.

In summary, the findings indicated that objective early-life environmental stressors directly or indirectly influenced adolescents’ life history strategies, characterized by shorter future orientation and increased risk-taking tendencies. Furthermore, perceived environmental unpredictability and parental warmth mediated the impact of socioeconomic status on adolescents’ risk-taking behaviors. Lastly, while biological sensitivity moderated the effect of mortality cues on perceived parental warmth, the sense of control did not mediate the relationship between early-life environmental stressors and risk-taking tendencies. Left-behind adolescents experienced a greater number of mobility and mortality cues, perceived higher unpredictability, and reported lower parental warmth compared to both single-left-behind and non-left-behind peers. Although general risk-taking tendencies did not differ significantly across the groups, left-behind adolescents displayed the highest risk-taking tendencies specifically in the health/safety domain.

## 4 Discussion

### 4.1 The environmental stresses that predict risk-taking

Rather than focusing solely on objective environmental stresses (33, 35), this study explored the relationship between two main clusters of environmental stresses—objective and subjectively perceived—and their effects on adolescents’ risk-taking tendencies. The objective cluster encompassed mortality cues, mobility cues, socioeconomic status, and being left-behind, while the subjective cluster included perceived unpredictability and perceived parental warmth. The correlations between these two clusters were not particularly strong in the final model, suggesting a need for further research to refine conceptualizations and measurements in this area.

The findings of this study indicate that adolescents may adopt a relatively faster life history strategy both cognitively and behaviorally in response to stressful environmental cues. Those who reported higher levels of objective environmental stresses and more intense subjective perceptions tended to display less future orientation and increased risk-seeking behaviors. According to life history theory, humans have evolved adaptive strategies to enhance fitness in response to varying environmental conditions (18, 42). Observable cues related to energetic conditions, harshness, and unpredictability trigger developmental adjustments along a slow-fast continuum, indicating different trade-offs (43).

In our study, environmental harshness was indicated by lower socioeconomic status and higher mortality cues, while unpredictability was represented by mobility cues and being left-behind. Participants who experienced these stressors exhibited reduced future orientation, and higher risk-taking tendencies. The indexes used to measure environmental stresses in this study expand on previous measures and provide a broader perspective on how these factors influence behavior (14, 17).

While ongoing debates exist about the relative impacts of harsh versus unpredictable childhood environments (16, 44, 45), this study found that both factors contributed unique aspects to explaining faster life history strategies. Interestingly, the indirect effects of subjective stresses explained more variance than the direct effects of objective stresses. Specifically, mortality cues were negatively associated with adolescent’ future orientation while positively correlated risk-taking across various domains. These results suggest that experiencing mortality salience adversely affects decision-making processes, emphasizing the importance of addressing perceptions and emotions related to such experiences in educational settings.

Mobility cues emerged as a positive predictor of risk-taking behaviors in the social domain, aligning with life history theory, which posits that individuals in unpredictable environments may adopt faster strategies to enhance offspring survival through increased reproductive efforts. Existing literature supports the notion that adolescents experiencing high residential mobility often exhibit riskier behaviors and earlier engagement in sexual activities (46, 47).

Regarding subjective stressful perceptions, perceived unpredictability was identified as the most significant factor predicting both future orientation and risk-taking across all domains. In contrast, perceived parental warmth was linked to risk-taking primarily in the ethical domain. This research underscores the necessity of considering subjective feelings about environmental conditions, as previous studies have predominantly emphasized objective measures. By integrating both objective and subjective environmental stresses, this study provides a more comprehensive understanding of the mechanisms through which early-life environments influence adolescents’ life history strategies.

### 4.2 The individual differences affecting risk-taking

Previous research has highlighted the interaction between biological sensitivity and adverse childhood environments in shaping socioemotional development among adolescents and adults (48–50). This study hypothesized that biological sensitivity would serve as a moderator between objective environmental stresses and subjective perceptions. However, the structural equation modeling (SEM) approach did not successfully fit the biological sensitivity construct within the current dataset, limiting the ability to directly assess this hypothesis. Therefore, the role of biological sensitivity remains an area for future exploration.

Despite the lack of direct analysis, it is clear that biological sensitivity may play a significant role in how individuals perceive and react to environmental stressors. Individuals with higher biological sensitivity may experience environments differently, suggesting that they require tailored approaches for coping with adversity. Understanding and nurturing this sensitivity rather than attempting to “fix” it could foster healthier development. Providing support to build specific coping strategies can be more beneficial than neglecting or blaming high sensitivity.

In addition to biological sensitivity, the concept of sense of control was also examined as a potential mediator (30–32). However, the hypotheses regarding the interactions between sense of control and environmental adversity in shaping life history strategies were not supported by the findings in this study. One reason for this may be the limited scope of the sense of control measure, which consisted of only four items and may not comprehensively capture the nuances of this construct. Although sense of control was positively associated with future orientation and risk-taking, indicating that adolescents with a higher sense of control may view the outcomes of their risk-taking behaviors as more manageable, the direct correlation with life history strategy remains unclear.

Nevertheless, previous studies have established a positive relationship between a sense of control and future orientation, as well as their independent effects on delinquent behavior (51). These findings suggest that enhancing adolescents’ sense of control may empower them to plan better for their futures and understand the potential consequences of their behaviors.

### 4.3 The case of left-behind adolescents

For children and adolescents, parents play a crucial role in shaping their understanding of the macro-environment they inhabit (52, 53). Left-behind adolescents often endure inadequate emotional and material support. Prolonged separation from parental figures can foster a sense of insecurity about the world and a feeling of having nothing to lose. Consequently, these adolescents may engage in risk-seeking behaviors, prioritizing immediate gratification over long-term planning.

In this study, the left-behind status was identified as an indicator of environmental unpredictability. Particularly those with neither parent presence face more challenges and instability. This group experienced heightened mobility and mortality cues compared to their non-left-behind peers. As a result, left-behind adolescents perceive greater unpredictability and exhibit lower levels of perceived parental warmth than single-left-behinds, who, in turn, show lower warmth than non-left-behinds.

Our findings emphasize the urgent need for targeted educational policies. We call on migrant working parents to attempt altering their living arrangements with their children. If migration is unavoidable, maintaining the presence of at least one parent can significantly mitigate the negative effects associated with parental absence. Furthermore, regular communication through phone calls, video chats, and letters can help bridge the gap created by physical separation. Research indicated that secure parent-child attachment can alleviate symptoms of depression in left-behind children (54, 55). While parental migration may provide financial support, it often fails to address the broader adverse effects on the well-being of left-behind adolescents (56). Additionally, cultivating supportive community networks is vital (55, 57), as positive interactions with teachers and peers serve as protective factors for the mental health of left-behind children.

Despite no significant differences in future orientation or overall risk-taking scores among adolescents of various left-behind statuses, those who are left behind tend to engage in more health and safety risk-taking behaviors than their peers. This finding poses particular concern, as left-behind adolescents overwhelmed by life stressors may neglect their physical health. Research by Li et al. (58) indicates that left-behind children without parental presence are 20% more likely to experience illness. A meta-analysis conducted by Wu et al. (59) identified learning anxiety, social anxiety, and physical symptoms as prevalent mental health issues among left-behind children. Given these challenges, educators should pay close attention to left-behind children’s health-related risk behaviors, including alcohol consumption, smoking, engaging in dangerous activities, and self-injury.

Previous studies have primarily focused on comparative analyses, revealing the greater academic, emotional, and behavioral difficulties faced by left-behind children compared to their non-left-behind counterparts (60–62). However, few have delved into the psychological mechanisms regarding the impacts of being left behind. While negative outcomes are well-documented, merely outlining these does not lead to effective preventive or remedial strategies. This study offers a new perspective on the direct and procedural effects of the left-behind status on adolescents, enabling relevant recommendations aimed at improving support for this vulnerable group. Future research should explore potential buffering factors, such as the specific caregivers with whom adolescents reside, the frequency of contact with absent parents, and the age at which parental absence first occurred.

### 4.4 Contributions and implications of the study

This study contributes significantly to a new evolutionary developmental model that enhances our understanding of risk-taking behaviors in adolescents, particularly in the context of rural China. The findings contribute to the growing body of literature linking early-life environmental stresses to major life outcomes, emphasizing how different forms of adversity influence risk-taking tendencies in later life.

Empirically, this study identifies environmental stresses faced by left-behind children and adolescents in Mainland China, presenting them as a vulnerable group with increased rates of risk-taking behaviors, particularly in health and safety domains. Unlike previous research that mainly described the status of left-behind children, this study investigates the mechanisms through which parental absence affects these adolescents.

The implications of this research extend to intervention policies aimed at supporting left-behind children and adolescents, especially in China and other developing countries facing similar migration issues. It stresses the importance of addressing both macro and micro-level strategies to reduce the prevalence of left-behind children and to mitigate risk-taking behaviors in healthier ways.

While the present study reveals significant associations between early-life stress and risk-taking behaviors of adolescents, it is important to acknowledge certain limitations that will guide our direction for future research. Methodologically, the cross-sectional design restricts our ability to draw causal conclusions. Future research will benefit from a longitudinal approach, which is essential for exploring the dynamics of risk-taking tendency over time in relation to early-life stress. Also, relying on self-reported measures may introduce bias into our study. Therefore, incorporating a multi-method assessment strategy, potentially including objective measures such as behavioral observations and experimental outcomes, will enhance the reliability and validity of our findings in the future.

Our sample, derived from a single county in rural China, may not fully represent the broader population. To enhance the generalizability, future studies will aim to include a more diverse and extensive sample of adolescents from various regions. Furthermore, many of the measurement scales used in this study were originally developed in Western cultures, necessitating further validation for use in the Chinese context. Future research should consider incorporating more indigenous measures to comprehensively assess early-life environmental stresses and their long-term effects.

Despite these limitations, our study meaningfully contributes to the existing literature by elucidating the importance of considering early-life environmental stress when examining risk-taking behaviors in adolescents. The insights gained from this research highlight the need for ongoing research to explore the complexities of these relationships. As we move forward, the incorporation of longitudinal data, objective measures, and a diverse sample will not only strengthen our conclusions but also broaden the applicability of our research findings.

## Data Availability

The original contributions presented in the study are included in the article/supplementary material. Further inquiries can be directed to the corresponding author.
